# *Twist1*-Haploinsufficiency Selectively Enhances the Osteoskeletal Capacity of Mesoderm-Derived Parietal Bone Through Downregulation of *Fgf23*

**DOI:** 10.3389/fphys.2018.01426

**Published:** 2018-10-15

**Authors:** Natalina Quarto, Siny Shailendra, Nathaniel P. Meyer, Siddharth Menon, Andrea Renda, Michael T. Longaker

**Affiliations:** ^1^Hagey Laboratory for Pediatric Regenerative Medicine, Department of Surgery, Stanford University, School of Medicine, Stanford, CA, United States; ^2^Dipartimento di Scienze Biomediche Avanzate, Universita’ degli Studi di Napoli Federico II, Naples, Italy

**Keywords:** *Twist1*, haploinsuffiency, *Fgf23*, downregulation, enhancement, osteoskeletogenesis

## Abstract

Craniofacial development is a program exquisitely orchestrated by tissue contributions and regulation of genes expression. The basic helix–loop–helix (bHLH) transcription factor Twist1 expressed in the skeletal mesenchyme is a key regulator of craniofacial development playing an important role during osteoskeletogenesis. This study investigates the postnatal impact of *Twist1* haploinsufficiency on the osteoskeletal ability and regeneration on two calvarial bones arising from tissues of different embryonic origin: the neural crest-derived frontal and the mesoderm-derived parietal bones. We show that *Twist1* haplonsufficiency as well *Twist1*-sh-mediated silencing selectively enhanced osteogenic and tissue regeneration ability of mesoderm-derived bones. Transcriptomic profiling, gain-and loss-of-function experiments revealed that *Twist1* haplonsufficiency triggers its selective activity on mesoderm-derived bone through a sharp downregulation of the bone-derived hormone *Fgf23* that is upregulated exclusively in wild-type parietal bone.

## Introduction

The mammalian skull vault is a structure formed by five intramembranous flat bones: the paired frontal and parietal bones and the unpaired interparietal bone (Morriss-Kay, 2001). The adjacent osteogenic fronts of these intramembranous bones form the sutures, which accommodate the growth of skull vault and underlying brain. The morphogenesis of the skull vault is accomplished in two phases: the first is defined by the genesis, migration and early specification of skeletogenic precursor cells. The second the differentiation of the skeletogenic mesenchymal precursor cells and subsequent appositional growth of the bones. Calvarial bones arise from two embryonic tissues, namely, the neural crest and the mesoderm. The mixed developmental origin of the mammalian skull vault has been clearly defined by employing a Wnt1-Cre/conditional LacZ reporter R26R double transgene mouse tracing exclusively neural crest cells, and showing that the frontal and squamosal bones are neural crest-derived, while the parietal bone is of mesoderm-derived (Jiang et al., 2002). Later, Yoshida et al. (2008) elegantly validated the same dual embryonic origin of the frontal and parietal bones, by performing the reciprocal study using the Mesp1-Cre transgene combined with R26R, which uniquely identified mesodermal cells.

Recently, important progress has been accomplished in our understanding of how enhanced activation of specific signaling pathways contributes to differences in the osteogenic capacity and bone repair observed between neural crest-derived and mesoderm-derived calvarial bones. Our previous investigations unveiled that disparate embryonic tissue origin translates into regional differences in osteogenic and regenerative ability of parietal and frontal bones. Furthermore, our findings revealed that skeletal differences observed between the two calvarial bones are controlled by the integration of multiple and converging osteogenic signaling pathways differentially activated between frontal and parietal bones (Senarath-Yapa et al., 2013). We identified an enhanced activation of endogenous FGF, BMP and canonical Wnt (cWnt) signaling exclusively in the neural crest-derived frontal bone (Quarto et al., 2009a,b; Li et al., 2015), whereas enhanced activation of TGF-β signaling is found in the mesoderm-derived parietal bone (Li et al., 2013; Senarath-Yapa et al., 2016).

During the development of skeleton and bone tissue ossification, multiple morphogenetic growth factor and hormone signaling pathways impact transcriptional regulators to trigger the osteogenic phenotype (Karsenty, 2008; Karsenty et al., 2009; Karsenty and Wagner, 2002). Twist1 is an evolutionarily highly conserved transcription factor (Murre et al., 1989; Miraoui and Marie, 2010) originally identified in *Drosophila* (Thisse et al., 1987; Leptin, 1991). This transcription factor plays extensive roles in skeletogenesis, directing embryonic skeletal patterning, fetal skeletal development, and bone remodeling (Howard et al., 1997; Rice et al., 2000; Yousfi et al., 2001; O’Rourke and Tam, 2002; Bialek et al., 2004; Marie et al., 2008; Wu et al., 2008; Bildsoe et al., 2009). Studies in *Drosophila* development showed that *Twist* is expressed in mesodermal and cranial neural crest (CNC) cells (Thisse et al., 1988) and plays a key-role in mesoderm-specification and myogenesis (Thisse et al., 1987; Leptin, 1991; Baylies and Bate, 1996; Cripps and Olson, 1998). Moreover, the expression pattern of *Twist1* in mouse suggests that this transcription factor regulates genes controlling the specification and the differentiation of cranial mesenchyme (Bildsoe et al., 2009). *Twist1* is also found expressed *in vivo* in mesoderm-derived osteoblast progenitors as well as *in vitro* in calvaria murine osteoprogenitors (Murray et al., 1992) suggesting that Twist1 may play a key-regulator role during osteoblast differentiation. In humans heterozygous loss of *TWIST1* function causes the Saethre–Chotzen syndrome. Individuals affected by this genetic condition have premature fusion of coronal suture, known as craniosynostosis. (Howard et al., 1997; Marie et al., 2008). Like humans, *Twist1* mutant mice also are affected by coronal suture craniosynostosis (el Ghouzzi et al., 1997; Carver et al., 2002).

The current study focuses on the role of transcription factor Twist1 and its interplay with osteoskeletal phenotype of calvarial bones of different embryonic tissue origin. Our results indicate that *Twist1* haploinsufficiency and/or *Twist1* shRNA silencing enhanced the osteoskeletal phenotype of mesoderm-derived parietal bone and osteoblasts. This selective osteoskeletal induction triggered by *Twist1* haploinsufficiency is mediated by a significant downregulation of *Fgf23*, a crucial player in balancing homeostasis of bone mineralization (Shimada et al., 2001; Larsson et al., 2004; Shimada et al., 2004). Our findings highlight new functional aspects of *Twist1* haploinsufficiency in the context of calvarial bone skeletogenesis/regeneration and point to Fgf23 as putative target of Twist1 activity.

## Materials and Methods

### Animals Ethical Approval

Experiments using animals were carried out following Stanford University Animal Care and Use Committee guidelines. All research has been approved by Stanford APLAC, with a number of protocol #9999, following approved guidelines by the Stanford University’s Institutional Review Board. *Twist1*^+/−^ mice were purchased from Jackson Laboratory (Bar Harbor, ME, United States). Genotyping was performed as previously described (Chen and Behringer, 1995; Behr et al., 2011a) CD-1 mice were from (Charles Rivers Laboratories, Inc., Wilmington, MA, United States).

### Osteoblasts Primary Cultures

Frontal and parietal calvarial bones were harvested from postnatal day 21 (pN21) *Twist1* haploinsufficient and wild-type mice (from the same litter) using a dissecting microscope. After meticulous removal of periosteum and dura mater from the calvaria skull, frontal and parietal bones were dissected free from surrounding sutures. Bones were then mechanically minced and digested using 0.2% dispase II and 0.1% collagenase A (Roche Diagnostics, Indianapolis, IN, United States) in serum-free medium in a shaker water bath at 37°C. Digestion was carried out six times (10 min each) as previously described (Quarto et al., 2009b; Li et al., 2013). The first two digestions were discarded. The last four digestions were pooled, centrifuged, and resuspended in non-inductive growth media α-minimal essential medium (α-MEM)-GlutaMax supplemented with 10% FBS and 100 IU/ml Penicillin/Streptomycin (GIBCO, Invitrogen, Carlsbad, CA, United States) as previously reported (Quarto et al., 2009b).

### Osteogenic Differentiation Assay

For the osteogenic assay osteoblasts were seeded in six-well plates at 5 × 10^5^ per well. After 24 h, cells were cultured in inductive osteogenic medium (day 0) comprising of α-MEM-GlutaMax supplemented with 10% FBS, 100 IU/ml penicillin, 100 IU/ml streptomycin, 10 mMβ-glycerophosphate, and 100 μg/ml ascorbic acid (Sigma-Aldrich, St. Louis, MO, United States). Experiments were carried out using only first and second passage cells. Osteoblasts were collected at different time points (d0, d3, d10, and d28) and RNA isolated for the analysis of osteogenic markers. Extracellular matrix mineralization and bone nodules formation were analyzed by Alizarin red and/or von Kossa staining procedures as described previously (Quarto et al., 2009b, 2015).

### RNA Isolation, Reverse Transcription (RT), and qPCR Analysis

Total RNA was isolated either from osteoblasts or frontal and parietal bones using TRIzol method according to the manufacturer’s protocol (Invitrogen, Carlsbad, CA, United States). Upon DNAse I (Ambion; Austin, TX, United States) treatment to clear genomic DNA, RNA was reverse transcribed using a SuperScript III First-Strand kit (Invitrogen, Carlsbad, CA, United States) as previously described (Quarto et al., 2008, 2009b). Osteogenic gene expression profile was examined by RT-qPCR. The relative mRNA level in each sample was normalized to its *Gapdh* content. Values are provided as relative to *Gapdh* expression. The results are presented as means ± SD of triplicate.

Primers sequence are as follows: m*Twist1*-Forward: 5′-TTCAGACCCTCAAACTGGCG-3′; m*Twist1*-Reverse: 5′-CTGGGAATCTCTGTCCACGG-3′; m*Fgf23*Forward: 5′-CGAGGAAAGAGTTCACGCCT-3′; m*Fgf23*-Reverse: GCGTCTTTCTTCGACTTGCC; m*Osx*-Forward: 5′-AGCGACCACTTGAGCAAACAT-3′; m*Osx*-Reverse; 5′-GCGGCTGATTGGCTTCTTCT-3′. Annealing temperature at 58°C. Primers sequence for *Bglap* (osteocalcin) and *Gapdh* and PCR conditions were reported previously (Quarto et al., 2009b). The results are presented as means ± SD of triplicate.

### Creation of Calvarial Defects

Animal experiments were performed in accordance with Stanford University Animal Care and Use Committee guidelines. Briefly, after anesthesia with an intraperitoneal injection of ketamine 100 mg/kg + xylazine 20 mg/kg + acepromazine 3 mg/kg and disinfection of the surgical site of the mice, an incision was made laterally to the sagittal midline to expose the frontal and parietal bones. The pericranium was removed using a sterile cotton swab. Using diamond coated trephine bits and saline irrigation, bilateral full-thickness calvarial defects (2-mm in diameter) were created in the non-suture associated right frontal and left parietal bones of pN21 mice (wild-type = 5 mice, *Twist1*^+/−^ *n* = 5 mice). Meticulous care was taken in order to protect the underlying dura mater or neighboring cranial sutures (Quarto et al., 2009b; Behr et al., 2011b, 2012).

### Assessment of Calvarial Healing Rate

The rate of healing μCT-scanning was performed as previously described (Behr et al., 2011b, 2012). Briefly, mice were scanned with a high-resolution MicroCAT II scanner (ImTek Inc., Knoxville, TN, United States) with an X-ray voltage of 80 kVP and an anode current of 450 μA. X-ray data reconstruction was performed with Cobra EXXIM (EXXIM Computing Corp., Livermore, CA, United States), and Micro View Software (GE Healthcare, Chicago, IL, United States). Each mouse was scanned with a CT-phantom, which is used to calibrate each scan. The precise threshold for regenerating calvarial bone was determined equivalent to 510 Houndsfield Units. The rest-defect areas were then determined with the Magic Wand Tool in Photoshop (Adobe Systems, San Jose, CA, United States). The area of the calvarial defects was evaluated by quantifying pixels in the defect. Percentage healing was then determined by dividing the rest defect area by the mean of defect size 1 day postoperatively. Mice were scanned 24 h post-surgery (time 0) and at week 1 2, 3, 4, 5, 6, and 7. For statistical analysis was used the Mann–Whitney test. A ^∗^*p*-value < 0.05 was considered statistically significant. For determining bone mineral density (BMD), standardized regions in the uninjured frontal and parietal bones were chosen and analyzed with the BMD tool in MicroView. The threshold range was set between 900 and 3500 voxels. The software automatically performed data analysis and calculations. Measurements were performed on four mice for each strain.

### Silencing of *Fgf23* and *Twist1* in Osteoblast Cells

Osteoblasts (pN21) were plated onto 12-well plates to 50% confluency 1 day before transduction. Cells were transduced with either *Fgf23*shRNA lentiviral particles (sc-39487-V) or *Twist1*shRNA lentiviral particles (sc-38605-V) Santa Cruz Biotechnology, Santa Cruz, CA, United States) and shRNA (scramble) lentiviral particles-A (sc-108080) was used as control (Santa Cruz Biotechnology, Santa Cruz, CA, United States). Transductions were performed according to the manufacturer’s protocol and as previously reported (Quarto et al., 2015). The efficiency of transduction was monitored 48 h after co-transduction using copGFP Control Lentiviral Particles (sc-108084), Santa Cruz Biotechnology, Santa Cruz, CA, United States) and found to be 85–87%. Two days later, the transduced cells underwent to puromycin selection (5 μg/mL) for 5 days. Efficiency of *Fgf23* and/or *Twist1* knockdown was assessed either at gene expression level by RT-PCR analysis or at protein level by immunoblotting analysis. Transduced cell were cultured in presence of puromicin thoroughly all the time of experiments.

### Immunoblotting Analysis

Immunoblotting analysis was performed using the following primary rabbit antibodies: rabbit anti-Twist1 (H-8: sc-15393) (dilution 1:200, Santa Cruz Biotechnology, Santa Cruz, CA, United States), rat monoclonal anti-FGF23 (MAB2629) (dilution 1:200, R&D System, Minneapolis, MN, United States) and anti-*β*-Actin (ab8227) (dilution 1:5,000; Abcam, Cambridge, MA, United States). Cell lysate proteins (40–80 μg) were resolved by NuPAGE 4–12% bis-Tris-HCl sodium dodecyl sulfate-polyacrylamide gel (Novex, Life Technologies, Carlsbad, CA, United States). Proteins were transferred to a polyvinylidene fluoride membrane (Bio-Rad, Inc., Hercules, CA, United States) and probed with specific antibody. Horseradish peroxidase-conjugated secondary anti-rabbit (7074S) and anti-rat antibodies (7077S) (dilution 1:2000; Cell Signaling, Danvers, MA, United States) were used. Immunoblotted proteins were visualized by enhanced chemiluminescence (Amersham Biosciences, Buckinghamshire, United Kingdom).

### Preparation of Cell-Conditioned Media and FGF23 Enzyme-Linked Immunosorbent Assay

Cell-media were collected from subconfluent cells cultured for 48 h in growth medium supplemented with 2% FCS. Media were then concentrated 50-fold using Centricon filters (3000 NMWL, Millipore Corporation, Billerica, MA, United States). Collection and concentration of the media were carried out at 4°C. The volume of each conditioned medium was normalized by cell numbers. Protein concentration was determined by BCA protein assay (Pierce Biotechnology, Rockford, IL, United States). Media were analyzed for FGF23 concentrations by enzyme-linked immunosorbent assay (ELISA) using Quantakine mouse FGF23 kit (R&D Systems, Minneapolis, MN, United States), according to the manufacturer’s instructions. Photometric detection was done with an ELISA reader at 370-nm wavelength. Each sample was run in triplicate. The results are the mean ± SD of three independent experiments.

### Bulk RNA-Sequencing (RNA-Seq)

RNA-seq was performed by the Stanford Functional Genomics Facility (SFGF) core at Stanford University^1^. Total RNAs isolated from wild-type and *Twist1*^+/−^ frontal and parietal bones (mice = 12/each strain) were purified using a Qiagen MiRNeasy Kit (Cat # 217004, Qiagen, Inc, Valencia, CA, United States), according to the manufacture’s instructions. Libraries were constructed using KAPA Stranded mRNA-Seq Kit (Cat # KK8420, Kapa Biosystem, Wilmington, MA, United States) following the manufacture’s instructions. Briefly, magnetic oligo-dt beads were used for poly (A) capture, followed by first strand synthesis using random primers, second strand synthesis, converting cDNA:RNA hybrid to double-stranded cDNA and incorporating dUTP into the second cDNA strand. Subsequently, A-tailing to add dAMP to the 3′-ends of the dscDNA library fragments was performed. An adapter ligation was carried out, where dsDNA adapters with 3′dTMP overhangs were ligated to the A-tailed library insert fragments. Library amplification was then performed to amplify library fragments carrying appropriate adapter sequences at both ends using high-fidelity, low-bias PCR. Amplified libraries were run on bio-analyzer to determine the library fragment size distribution and to detect the presence of excessive adapter dimer molecules using the High Sensitivity DNA Assay (Cat # 5067-4626 Agilent Technologies, Santa Clara, CA, United States). The quantified libraries were then pooled at equal nanomoles of individual libraries. After final QC the pooled libraries were sequenced on Illumina Next-Seq (Illumina, San Diego, CA, United States).

### RNA-Seq Analysis

FastQC 0.11.2^2^ from the Babraham Institute was used to perform quality control checks on raw sequence data. The mouse reference genome (mm9, NCBI Build 37) was obtained from the UCSC Genome Browser download site. The corresponding reference annotations (GTF) were obtained from the iGenomes site hosted by Illumina. The splice-aware aligner STAR [version 2.4.2a, (doi: 10.1093/bioinformatics/bts635)] was used to align reads to the mouse reference genome. Cuffdiff (Cufflinks version 2.2.1) was used for gene expression quantification (in FPKM) and for detection of differentially expressed genes based on the BAM alignments from STAR. The cummeRbund R package (version 2.12.1 with R version 3.2.2) was used for visualization and sample clustering. Venn (version 0.8.4) was used to generate dynamic Venn diagrams^3^ for differential gene expression based on the outputs from Cuffdiff. Hierarchical clustering of the gene expression data was done on log_2_ (FPKM + 1) values using the Cluster (version 3.0) software and the output (CDT, GTR, and ATR files) was visualized as heatmaps using Java TreeView (version 1.1.6r4)^4^. GSEA (version 2.2.0, DOI: 10.1073/pnas.0506580102) was used to perform gene set enrichment analysis to identify statistically significant gene sets showing concordant differences between two biological states. Each replicate corresponding to wild-type and *Twist*^+/−^ frontal and parietal bones had, on an average, 63 million single-end reads of length 75 bp. Mapping of reads to the mm9 reference genome using STAR aligner resulted in, on an average, 74% uniquely mapped reads and 15% multi-mapped reads resulting in 89% mapped reads overall. Transcript abundances in terms of FPKMs were determined using Cuffdiff and the large amount of data produced from the Cuffdiff RNA-Seq differential expression analysis were then visualized using the cummeRbund R package as follows. Density plots were examined to assess the distributions of FPKM scores across samples

### Calcein Incorporation

**FIGURE 1:**
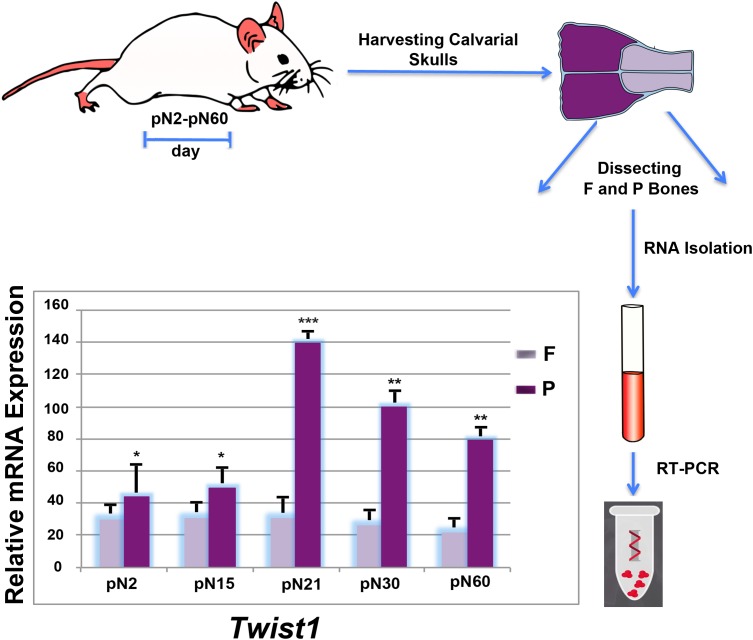
Differential postnatal expression of *Twist1* in frontal and parietal bones. Time-course RT-qPCR analysis performed on frontal and parietal bone tissues (*n* = 10) revealed a differential expression of *Twist1* with higher levels in parietal bone. The relative mRNA level in each sample is normalized to housekeeping gene *Gapdh*. Values are presented as relative to *Gapdh* expression. Values: ^∗^*p* ≤ 0.05, ^∗∗^*p* ≤ 0.01, and ^∗∗∗^*p* ≤ 0.001.

To analyze the formation/mineralization rate of wild-type and *Twist1^+^*^/−^ frontal and parietal bones, we harvested skulls from pN2 mice and cultured for 2 days in complete a-MEM medium supplemented with calcein (1 μg/ml) (Millipore-Sigma, St. Louis, MO, United States). Where requested mouse recombinant FGF23 protein (100 ng/ml, R&D System, Minneapolis, MN, United States) was added to the cultures. Specimens were fixed in 10% neutral buffered formalin overnight at 4°C, embedded in OCT and cut into 8-μm section. Images of frozen sections were captured under a fluorescence microscope (Leica DMI 4000B) using the same exposure time, and the rate of fluorescence measured using J image densitometer. Sixty images selected randomly from 3 bones (for each group) were analyzed and data presented as average fluorescence intensity. Experiments were performed twice. Of note, technical aspects arising from this type of experiment dictated the choice of pN2 mice. Specifically, in order to preserve the calcein incorporation into the bony tissue, the specimens were not decalcified by EDTA, because this treatment would dissolve the calcein incorporated into the bone. Therefore, to be able to cut the bone into sections the tissue has to be soft and skull of pN2 meets this need. Of note, at pN2 *Twist1* gene is already differentially expressed between frontal and parietal bones (see **Figure 1**).

### Statistical Analysis

Data are presented as mean-SD of three independent samples. Statistical comparisons between groups were done using a two-tailed Student’s *t* test, ^∗^*p* ≤ 0.05; ^∗∗^*p* ≤ 0.01; and ^∗∗∗^*p* ≤ 0.001 were considered significant.

## Results

### *Twist1* Is Differentially Expressed in Calvarial Bones of Different Embryonic Tissue Origin

While the spatio-temporal expression profile of *Twist1* during mouse embryonic development has been widely investigated (Chen and Behringer, 1995; Fuchtbauer, 1995; Stoetzel et al., 1995; Gitelman, 1997; Bildsoe et al., 2009), its postnatal expression pattern in bones of different embryonic tissue origin such as, the neural crest-derived frontal bone and paraxial mesoderm-derived parietal bone has not been described. Therefore, we began our study by profiling the postnatal *Twist1* expression in these two bones at different time points. The analysis obtained by RT-PCR revealed a distinct expression pattern between the frontal and parietal bones, which was characterized, starting from day pN2 by a significant increase of Twist1 expression in the mesodermal-derived parietal bone which reach a peak at day pN21, followed by downregulation at later time points. Conversely, in the neural crest-derived frontal bone *Twist1* expression was markedly lower and remained steady (**Figure 1**). This finding opens the question: does *Twist1* haploinsufficiency differently impact frontal and parietal bones? In order to address this question, we compared both, *in vitro* and *in vivo*, the osteoskeletal ability of frontal and parietal bones derived from haploinsufficient *Twist1* and wild-type mice.

### *Twist1* Haploinsufficiency Enhances *in vitro* Osteogenic Potential of Mesoderm-Derived

#### POb

Our previous studies demonstrated that neural crest-derived frontal osteoblasts (FOb) and mesoderm-derived parietal osteoblasts (POb) display a different osteogenic potential, with FOb differentiating more robustly than POb (Quarto et al., 2009b).

**FIGURE 2:**
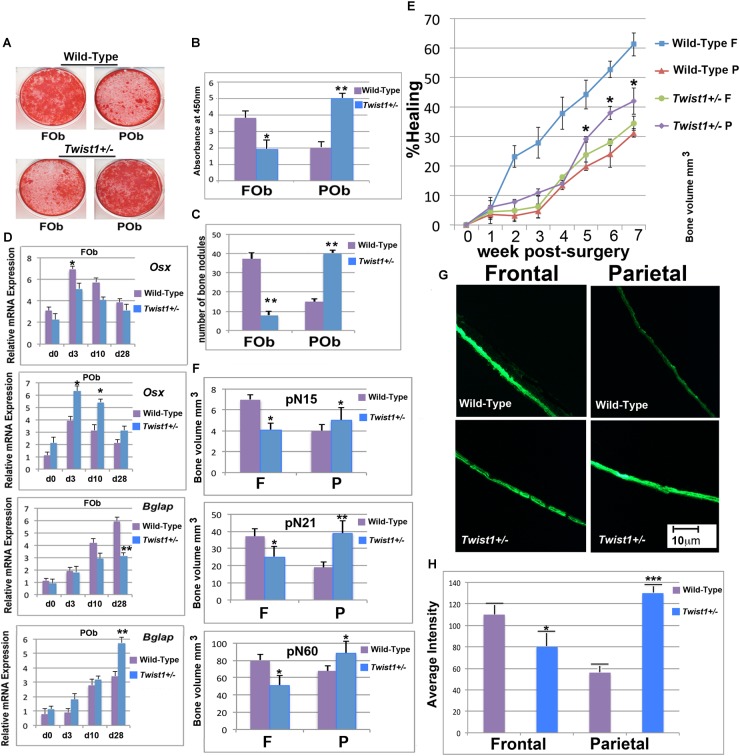
*Twist1* haploinsufficiency enhances the osteoskeletal potential of mesoderm-derived parietal bone. **(A)** FOb and POb cultured under osteogenic conditions for 28 days and stained with Alizarin Red. **(B)** Quantification of Alizarin Red staining revealing a more intense mineralization of the extracellular matrix in *Twist1*^+/−^ POb as compared to wild-type POb. **(C)** Number of large bone nodules formation identified by von-Kossa staining at osteogenic differentiation day 28. **(D)** Relative mRNA expression of early (*Osx*) and late (*Bglap*) osteogenic markers in wild-type and *Twist1*^+/−^ osteoblasts cultures. Results are representative of three experiments performed. **(E)** Time course of frontal and parietal bone healing defects, quantification of defect repair (*n*? = ?5/each bone) based on μCT results are analyzed using magic wand tool in Photoshop after standardization of threshold for regenerating calvarial bone. Results are representative of two independent experiments performed. Statistical analysis was performed using the Mann–Whitney Test. ^∗^*P* ≤ 0.05. **(F)** Quantification of bone thickness in uninjured wild type and *Twist1*^+/−^ Frontal and Parietal bones ^∗^*P* ≤ 0.05. **(G)** Bone formation in wild-type and *Twist1*^+/−^ frontal and parietal bones assessed by culturing skulls for 48 h in presence of calcein (1 μg/ml). **(H)** Quantification of calcein incorporation. Histomorphometric analysis of calcein accumulation was obtained using Image J64 densitometry analysis. Scale bar, 10 μm. Results are representative of three independent experiments performed. Values: ^∗^*p* ≤ 0.05, ^∗∗^*p* ≤ 0.01, and ^∗∗∗^*p* ≤ 0.001.

In the current study, we investigated whether a similar osteogenic profile would be observed in FOb and POb derived from haploinsufficient *Twist1* calvarial bones. Confluent wild-type and *Twist1*^+/−^ osteoblast cultures were incubated in osteogenic differentiation medium for 28 days followed by Alizarin red and von Kossa staining to assess for deposition of calcium into the extracellular matrix. As expected and previously described (Quarto et al., 2009b; Li et al., 2013) both staining methods revealed a robust mineralization of extracellular matrix and bone nodules formation in wild-type FOb compared to wild type POb (**Figures 2A–C**). Surprisingly, *Twist1*^+/−^ POb displayed greater osteogenic potential than wild-type POb, whereas *Twist1*^+/−^ FOb had less osteogenic potential than the wild-type counterpart (**Figures 2A–C**). This unique profile was also confirmed at molecular level by the expression of osteogenic markers specifically, the early marker osterix (*Osx*) and the late marker osteocalcin (*Bglap*) (**Figure 2D**). Thus, *Twist1* haploinsufficiency positively impacts the osteogenic ability of mesoderm-derived osteoblasts whilst impairing that of neural crest-derived osteoblasts.

### *Twist1* Haploinsufficient Enhances *in vivo* Bone Repair Ability Mesoderm-Derived Parietal Bone

Having observed an enhanced *in vitro* osteogenic potential of *Twist1* haploinsufficient POb as compared to wild-type POb, we sought to investigate whether a similar osteoskeletal phenotype would be observed *in vivo.* To this aim, we evaluated the calvarial healing ability of frontal and parietal bones in wild-type and *Twist1*^+/−^ mice. To explore *in vivo* healing, we introduced calvarial defects of 2-mm in diameter in the right frontal and left parietal bones of postnatal day 21 mice (*n* = 5). The healing rate of each defect was monitored for 7 weeks by microcomputed tomography (μCT). As previously reported (Quarto et al., 2009b; Li et al., 2015), μCT scanning of defects showed significantly less bone regeneration in wild-type parietal bone as compared to wild-type frontal bone (**Figure 2E**). Conversely, *Twist1* haploinsufficient parietal bone healed significantly better than wild type parietal bone to an extent similar to that of the wild type frontal bone (**Figure 2E**). Moreover, μCT analysis performed at different time points on uninjured frontal and parietal bones revealed differences in bone density, with *Twist1*^+/−^ parietal bone showing increased bone thickness as compared to wild-type parietal bone, and *Twist1*^+/−^ frontal bone having decreased bone density (**Figure 2F**). The latter observation found further support from calcein labeling experiments performed on *ex-vivo* skull explants derived from wild-type and *Twist1*^+/−^ mice. As shown in **Figures 2G,H**, *Twist1*^+/−^ parietal bones incorporated significant higher amount of calcein as indicated by the intensity of fluorescence and its quantification. This outcome demonstrates the ability of *Twist1*^+/−^ parietal bone to mineralize more robustly than wild-type, thus reflecting bone density properties. Taken together, results garnered from *in vivo* analysis mirrored those obtained from *in vitro*, supporting that *Twist1* haploinsufficiency leads to a significant enhancement of the osteogenic capacity of mesoderm-derived parietal bone while negatively impacting the neural crest-derived frontal bone.

### Transcriptomic Profile of *Twist1* Haploinsufficient Frontal and Parietal Bones

**FIGURE 3:**
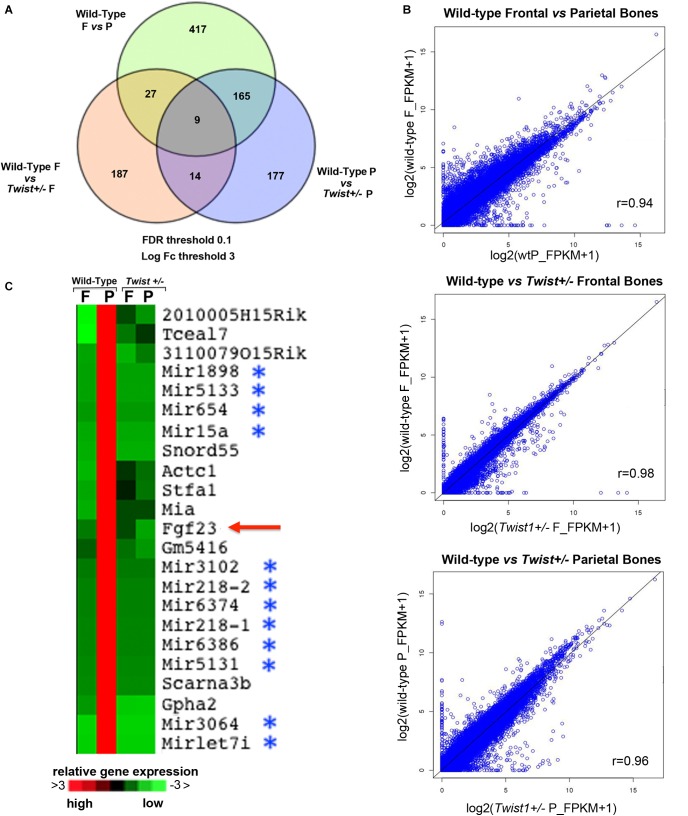
Transcriptomic profiles of wild-type and *Twist*^+/−^ frontal and parietal bones. RNA-Seq analysis to assess gene expression changes caused by *Twist1* haploinsufficiency in calvarial bone of pN21 mice revealed significant differences. **(A)** Venn diagram showing the comparison of top genes found differentially expressed (up and down regulated) in wild-type and *Twist*^+/−^ Frontal (F) and Parietal (P) bones for FDR < 0.1 and log_2_ (fold change) >3, with fold change as ratios of gene-level FPKM values. Their gene expression levels are affected ≥3 fold. **(B)** Scatterplot representation showing the fold change Scatter plots of gene expression of genes [based on log_2_ (FPKM + 1) expression values with each dot representing a gene]. In the bottom of each graph the pair-wise Pearson’s correlation (r) for all genes is shown. **(C)** Heatmap showing *Fgf23* as a component of a unique signature of genes that are exclusively upregulated in wild-type parietal bones. (upregulation in Red; downregulation in Green).

Next, we sought to investigate the molecular mechanism(s) through which *Twist1* haploinsuffiency could enhance the osteoskeletal ability of the parietal bone and derived osteoblasts. Our initial approach was to analyze the genomic profile of *Twist1*^+/−^ and wild-type frontal and parietal bones by bulk RNA-seq technique. Out of 24,015 mouse mm9 genes analyzed, this analysis revealed differential expression of 365 between wild-type parietal and *Twist1*^+/−^ statistically different (*p*-value < = 0.0001) with a fold change greater than three. Between wild-type and *Twist*^+/−^ frontal bones 217 genes were differentially expressed (*p*-value < = 0.0001) with a fold change greater than three. A greater differential gene expression was observed between wild-type frontal and parietal bones. Venn diagram and scatter plot analysis illustrate up or down regulated top genes with a fold change greater than three between the different bones (**Figures 3A,B**). Among the 365 genes differentially expressed between wild-type and *Twist1*^+/−^ parietal bones 310 were upregulated and 55 downregulated, while 233 were upregulated, and 4 downregulated between wild-type and *Twist1* frontal bones. The wild-type frontal and *Twist1*^+/−^ bones were the most closely correlated with a Pearson correlation coefficient (*r* = 98), than wild-type parietal and *Twist1*^+/−^ bones (*r* = 96) (**Figure 3B**). Among the genes differentially expressed between *Twist1*^+/−^ and wild-type parietal bones, we found potentially of interest the *Fgf23* gene, which encodes a peptide hormone regulating bone mineralization and levels of serum phosphate (Shimada et al., 2001, 2004; Yamazaki et al., 2002; Riminucci et al., 2003; Larsson et al., 2004; Wang et al., 2008). We identified *Fgf23* as part of an unique signature of genes exclusively upregulated in wild-type parietal bones and significantly down-regulated in *Twist1*^+/−^ parietal bone with a fold change greater than six thus, representing one of the top genes significantly down-regulated. Therefore, we thought that it could be a candidate gene to investigate. In addition to *Fgf23*, in this signature were also identified clusters of microRNAs (**Figure 3C**).

### *Fgf23* Downregulation Is Intrinsic of Osteoblast Cells

**FIGURE 4:**
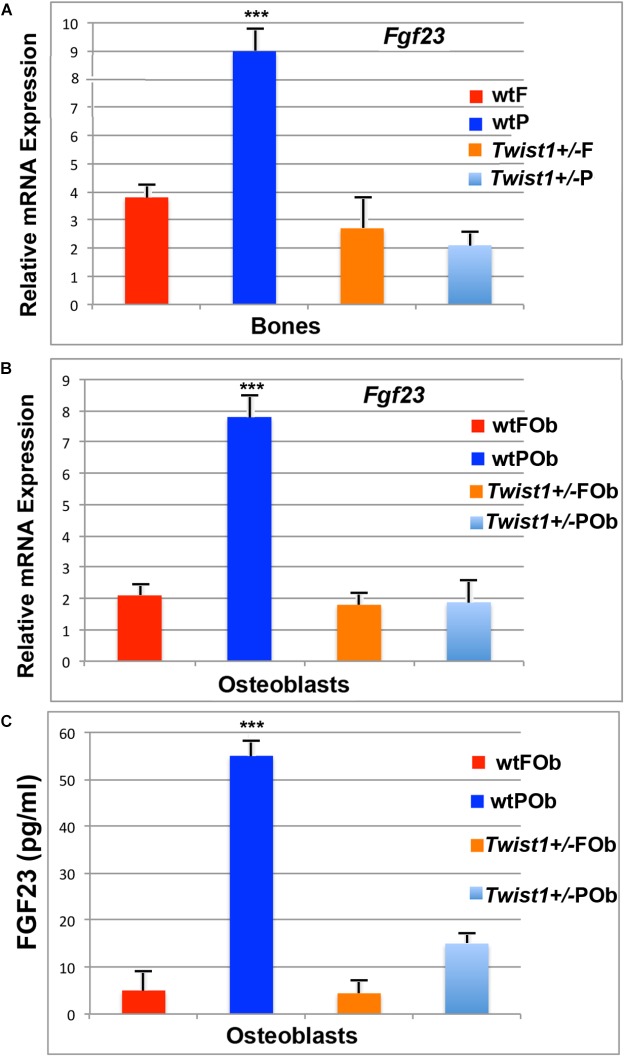
Validation of *Fgf23* in calvarial bones and derived osteoblasts. **(A)** Validation of *Fgf23* downregulation obtained by performing PCR analysis on bone tissues. **(B)** RT-qPCR analysis showing *Fgf23* down-regulation in osteoblast primary cultures (depleted of pericranium and dura mater- derived cells). **(C)** ELISA assay performed on media collected from osteoblast cells and concentrated by 20-fold. Elevated level of secreted FGF23 protein is detected in the medium of wild-type POb, whereas in *Twist*^+/−^ POb protein levels are sharply decreased to values similar to that of wild-type and *Twist*^+/−^ FOb. Value: ^∗∗∗^*p* ≤ 0.001.

As a first step, prior to investigating the potential involvement of *Fgf23* downregulation in enhancing POb osteogenic differentiation, we validated its differential expression by PCR analysis in bone tissues on which bulk RNA-seq analysis was performed. This analysis confirmed a significant downregulation of *Fgf23* in *Twist1*^+/−^ parietal bone as compared to wild-type (**Figure 4A**), thus validating the data obtained from RNA-seq analysis.

Previous studied have reported that *Fgf23* is primarily expressed in osteoblasts and osteocytes (Riminucci et al., 2003; Mirams et al., 2004) and systemically circulates (Yamazaki et al., 2002). Additional PCR analysis performed on osteoblast cultures also revealed the same *Fgf23* expression pattern as previously identified by RNA-seq analysis in bone tissues. Similarly, *Fgf23* was found to be downregulated approximately seven-fold in *Twist1*^+/−^ POb as compared to wild-type POb (**Figure 4B**). This finding is of interest and indicates that the expression pattern of *Fgf23* and the downregulation initially observed in parietal bones comprising of pericranium, bone plate and dura-mater, is intrinsic of osteoblast cells which were derived from calvarial bones depleted of pericranium and dura-mater tissues. Consistent with this finding, ELISA detected high level of secreted FGF23 protein exclusively in wild-type POb, whereas, it was dramatically reduced in *Twist1*^+/−^ POb (**Figure 4C**). Indeed, the validation of our RNA-seq results by RT-PCR builds support to further investigate the potential functional role that *Fgf23* down-regulation may play in enhancing the osteogenic potential of *Twist1*^+/−^ POb.

### Exogenous FGF Protein Reduces the Osteogenic Profile of *Twist*^+/−^ POb

**FIGURE 5:**
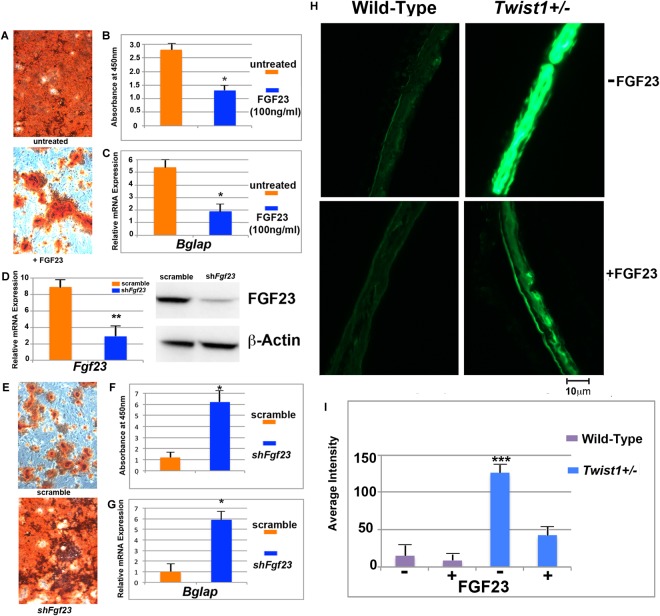
Inhibition of osteogenesis and bone mineralization by exogenous FGF23 protein. **(A)** Treatment with FGF23 protein (100 ng/ml) suppresses markedly osteogenic differentiation of *Twist*^+/−^ POb as revealed by Alizarin red staining performed at differentiation day 28. **(B)** Quantification of Alizarin red staining for the osteogenic assay shown in panel A **(C)** RT-qPCR analysis of the osteogenic marker osteocalcin (*Bglap*) confirms that FGF23 treatment abrogates the enhanced osteogenic of *Twist1*^+/−^ POb. **(D)** Validation of effective *Fgf23* silencing monitored by RT-qPCR (left panel) and immunoblotting analysis (right panel). **(E)** *Fgf23* silencing enhances the osteogenic capacity of wild-type POb mirroring that of *Twist1*^+/−^ POb as revealed by Alizarin red staining. **(F)** Quantification of Alizarin red staining for the osteogenic assay shown in panel **E**. **(G)** osteocalcin (*Bglap*) expression by RT-qPCR analysis confirms increased terminal differentiation in sh*Fgf23* POb compared to scramble POb. **(H)** Exogenous added FGF23 also decreases dramatically bone mineralization in parietal bone organ culture. Calcein was added to the cultures for 48 h. Immunofluorescence showing calcein accumulation into the bone. **(I)** Histomorphometric analysis of calcein accumulation. Scale bar, 10 μm. Results are representative of three independent experiments performed. Values: ^∗^*p* ≤ 0.05, ^∗∗^*p* ≤ 0.01, and ^∗∗∗^*p* ≤ 0.001.

Having confirmed the differential expression by PCR and protein analysis we sought to investigate the potential role Fgf23 might play in the osteogenic phenotype observed between wild type and *Twist1*^+/−^ POb. For this purpose, we performed a set of loss- and gain-of-function experiments. We began by treating osteoblasts with exogenously added FGF23 protein to assess whether this treatment would abrogate the increased osteogenic differentiation observed in *Twist1*^+/−^ POb. Outcomes from an osteogenic differentiation assay performed with or without FGF23 protein revealed a dramatic effect on *Twist1*^+/−^ POb. As shown in **Figures 5A,B**, Alizarin red staining detected a significant reduction of the extracellular matrix mineralization in FGF23 treated *Twist1*^+/−^ POb in comparison to untreated POb. These biochemical differences were further supported by the expression profile of late osteogenic marker *Bglap* (**Figure 5C**). Conversely, FGF23 treatment had little effect on *Twist1*^+/−^ FOb osteogenic profile which was already impaired by *Twist1* haploinsufficiency (**Supplementary Figures S1A–C**). Moreover, FGF23 treatment significantly impacted wild-type FOb, but had little effect on wild-type POb (**Supplementary Figures S1D–F**). The differential effect elicited by FGF23 on wild-type FOb and POb may reflect differences in the threshold level of endogenous FGF23 protein between FOb and POb, and therefore, their responsiveness. Taken together, these data strongly suggest that *Fgf23* downregulation may play a role in eliciting greater osteogenic capacity of *Twist1*^+/−^ POb.

### *Fgf23* Silencing Makes Wild-Type POb “*Twist1*^+/−^ POb-Like”

Next, we performed a counter part experiment seeking to investigate whether silencing of *Fgf23* in wild-type POb would potentiate their *in vitro* osteogenic ability mimicking that of *Twist1*^+/−^ POb. *Fgf23* silencing was successfully accomplished by transducing wild-type POb with sh*Fgf23* lentiviral particles. Upon puromicin selection, expression of *Fgf23* decreased approximately by 73% in sh*Fgf23* cells as compared to scramble transduced cells (**Figure 5D**, left panel). Immunoblotting analysis confirmed decrease of FGF protein (**Figure 5D**, right panel). Then, we performed an osteogenic differentiation assay to evaluate the osteogenic ability of sh*Fgf23*POb in comparison to scramble POb. The analysis provided clear evidence on the key role that downregulation of *Fgf23* plays in enhancing the osteogenic capacity observed in *Twist1*^+/−^ POb. This was indicated by a robust extracellular matrix mineralization as assessed by Alizarin red staining (**Figure 5E**) and its quantification (**Figure 5F**) as well as elevated expression level of *Bglap* (**Figure 5G**) in *Twist1*^+/−^ POb relative to scramble POb.

### Increased Calcein Incorporation in *Twist1*^+/−^ Parietal Bones

Fgf23 is a key player in regulating calcium homeostasis during bone cell differentiation and mineralization, and is abundantly expressed and secreted from immature and mature osteocytes within the bone matrix (Riminucci et al., 2003; Liao, 2013; Michigami, 2014; Guo and Yuan, 2015). Our results suggested that the osteoskeletal phenotype observed in *Twist1*^+/−^ parietal bone and POb is at least in part mediated by *Fgf23* downregulation. To further strengthen this observation we tested the ability of wild-type and *Twist1*^+/−^ parietal bone tissue explants in culture to incorporate calcein either in presence or absence of FGF23 protein. After 48 h in culture, incorporation of calcein into the bone explants was monitored by immunofluorescence. We observed that treatment with FGF23 dramatically decreased calcein labeling in *Twist1*^+/−^ parietal bone (**Figure 5H**, bottom panel) compared to untreated *Twist1*^+/−^ control bone (**Figure 5H**, top panel). The histomorphometric quantification analysis of calcein incorporation in untreated *Twist1*^+/−^ parietal bone showed a more intense immunofluorescence staining than FGF23 treated bone (**Figure 5I**). No significant differences were found between untreated and FGF23 treated wild-type parietal bones (**Figure 5H**, left panel, **Figure 5I**), this would reflect a lack of responsiveness by the cells due to high endogenous level of FGF23 and possible downregulation of its receptors. Taken together, these findings demonstrated that FGF23 treatment significantly inhibited bone mineralization on *ex vivo Twist1*^+/−^ parietal bone explants and further support its role in controlling the osteoskeletal properties between wild-type and *Twist1*^+/−^ parietal bones.

### *Twist1* Silencing Phenocopies *Twist1* Haploinsufficiency POb by Triggering Downregulation of *Fgf23*

**FIGURE 6:**
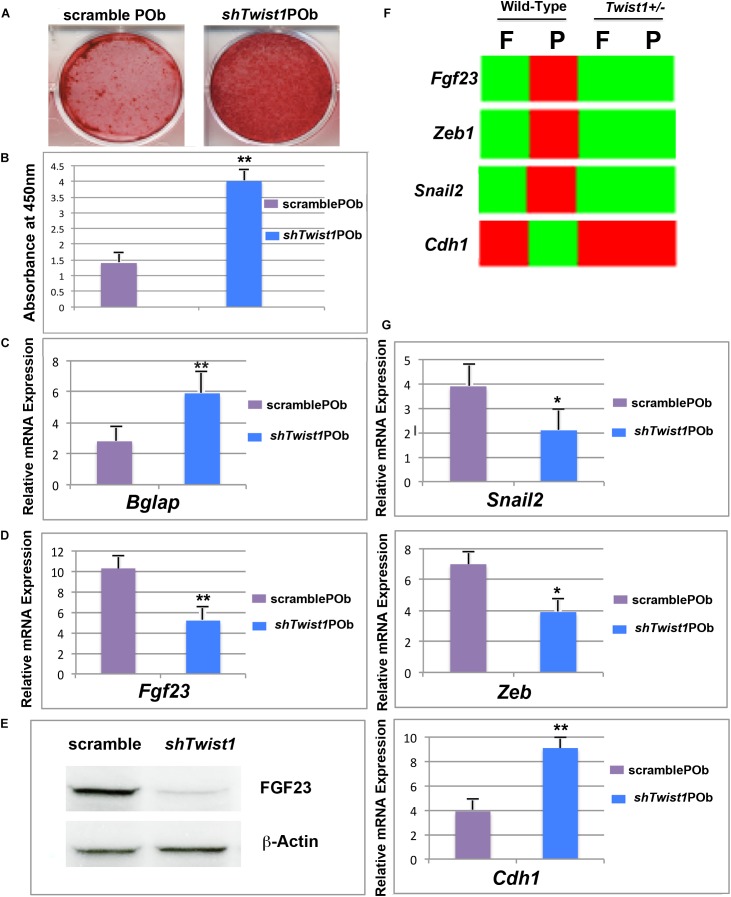
*Twist1* silencing phenocopies the osteoskeletal profile of *Twist1* haploinsufficiency in POb by downregulating *Fgf23*. **(A)** Osteogenic differentiation assay performed on sh*Twist1* POb revealed a robust extracellular matrix mineralization. **(B)** Quantification of Alizarin red staining for the osteogenic assay shown in panel **A**. **(C)** RT-qPCR analysis showing significant upregulation of *Bglap* as compared to scramble POb. **(D)** RT-qPCR analysis performed on stable transduced cells upon puromicin selection reveals that sh-mediated *Twist1* silencing triggers a significant downregulation of *Fgf23* expression. **(E)** Immunoblotting analysis using anti-FGF23 antibody confirms decreased FGF23 at protein level in sh*Twist1* POb compared to scramble POb. **(F)** Bulk RNA-seq analysis showing the effect of *Twist1* haploinsufficiency on some of its target genes such *Zeb*, *Snail1*, and *Cdh1* and comparison in wild-type versus *Twist1* haploinsufficiency parietal bones. **(G)** As assessed by RT-qPCR analysis a similar pattern is observed in stable transduced sh*Twist1* POb and scramble POb upon puromicin selection and culturing them. Values: ^∗^*p* ≤ 0.05, ^∗∗^*p* ≤ 0.01.

To gain further support to our findings, we next asked two questions: First, does silencing of *Twist1* in wild-type POb phenocopy *Twist1* haploinsufficient POb? Second, does it lead to downregulation of *Fgf23* as observed in *Twist1* haploinsufficient POb? To address these questions POb were transduced with sh*Twist1* or scramble lentiviral particles followed by puromicin selection. Silencing of *Twist1* was assessed by PCR and immunoblotting analyses (**Supplementary Figures S2A–C**). Stable transduced cells after puromicin selection were first analyzed for their osteogenic ability. As shown in **Figures 6A–C**, sh*Twist1* POb robustly mineralized their extracellular matrix and expressed significant higher levels of *Bglap* than scramble POb. Importantly, in addition to the enhanced osteogenic differentiation we observed a concomitant downregulation of *Fgf23* in sh*Twist1* POb. This was revealed either at the gene expression level by PCR analysis or at protein level by immunoblotting analysis performed on cultured sh*Twist1* POb (**Figures 6D,E**). Evidence of the functional effectiveness of *Twist1* silencing was provided by the expression analysis in sh*Twist1* POb of three known Twist1 target genes: *Zeb*, *Snail*, and *Cdh1* (Leptin, 1991; Rose and Malcolm, 1997; Kang and Massague, 2004; Peinado et al., 2007; Vesuna et al., 2008) and previously identified by our bulk RNA-seq as being differentially expressed between wild-type and *Twist1* haploinsufficient parietal bones (**Figure 6F**). Remarkably, PCR analysis performed on scramble and sh*Twist1* POb (**Figure 6G**) mirrored the expression pattern of *Zeb, Snail, Cdh1* previously observed in wild-type and *Twist1* haploinsufficient parietal bones. The above findings strongly suggest a direct involvement of *Twist1* haploinsufficiency in mediating the downregulation of *Fgf23*, which in turn confers a greater osteogenic ability to POb.

## Discussion

Previous studies have described Twist1 as a key-regulator of craniofacial development, however, the role of *Twist1* haploinsufficiency in skeletal development and regeneration of postnatal calvarial bones has not yet been fully delineated. Herein, we describe the impact of *Twist1* haploinsufficiency on two calvarial bones of different embryonic tissue: the neural crest-derived frontal bone and mesoderm-derived parietal bone. We provide evidence that *Twist1* haploinsufficiency enhances the osteoskeletal ability of the parietal bone compared to the corresponding wild-type bone by reaching a level similar to that of wild-type frontal bone. By performing bulk RNA-seq analysis we unveiled a distinct *Fgf23* upregulation in wild-type parietal bone, which is significantly suppressed by *Twist1* haploinsufficiency. Of note, the expression pattern of *Fgf23* observed in mesoderm-derived parietal bone is sustained from early to late postnatal stages (**Supplementary Figure S3**).

Several lines of evidence support the role of *Fgf23* downregulation in promoting a greater osteoskeletal capacity of *Twist1* haploinsufficient parietal bone. First, gain-of-function experiment showed that treatment with FGF23 protein significantly decreased osteogenic differentiation of *Twist1*^+/−^ POb as indicated by poor extracellular matrix mineralization and expression of specific osteogenic markers. Conversely, loss-of function by silencing *Fgf23* in wild-type POb enhanced their osteogenic differentiation mirroring the profile observed in *Twist1*^+/−^ POb. Second, bone density analysis and calcein labeling both detected a significant increased mineralization in *Twist1* haploinsufficient parietal bone where *Fgf23* is dramatically downregulated in comparison to wild-type. This finding was further corroborated by a complementary experiment showing that culturing *ex vivo Twist1* haploinsufficient parietal bones in presence of exogenous FGF23 protein led to decreased calcein-labeling, and therefore, mineralization to levels comparable to that of wild-type parietal bone. The latter result highlights a direct role of *Fgf23* downregulation in matrix mineralization of mesoderm-derived bones.

Impaired skeletal development is one major feature found in genetic disorders attributed to FGF23 overexpression (Liao, 2013). Mutation of this gene in humans causes autosomal dominant hypophosphatemic rickets (ADHR) (Shimada et al., 2002). Furthermore, high serum levels of FGF23 have been detected in patients affected by fibrous dysplasia (FD) of bone, which is a skeletal disorder with bone-forming cells failing to mature and aberrantly produce fibrous, or connective, tissue (Riminucci et al., 2003). Increased serum FGF23 has also been reported in X-linked hypophosphatemia (XLH) (Jonsson et al., 2003). Indeed, results garnered from our study further support FGF23 as a growth factor playing a central role in calcium homeostasis and acting on bone cell differentiation and mineralization.

**FIGURE 7:**
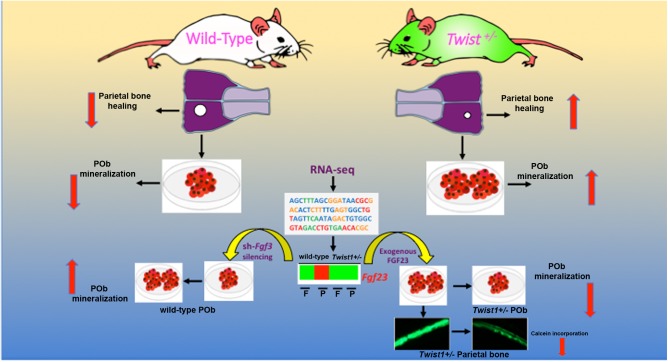
*Twist1*-haploinsufficiency-*Fgf23*-downregulation axis. Schematic representation of the molecular mechanism mediating the effect of *Twist1* haploinsufficiency in enhancing the osteoskeletal ability of mesoderm-derived parietal bone *via* downregulation of *Fgf23.*

Our findings unravel a novel role of *Twist1* haploinsuffiency in the context of calvarial bone differentiation and regeneration. Haploinsuffiency of this bHLH transcription factor triggers a sharp downregulation of *Fgf23* expression, which in turn enhances, selectively, the osteoskeletal ability of mesoderm-derived parietal bone, both *in vitro* and *in vivo* (**Figure 7**). Interestingly, *Twist1* haploinsuffiency negatively impacts the osteoskeletal property of the neural crest-derived frontal bone. The latter observation is not fully unexpected and recapitulates embryonic effects of loss of *Twist1* on CNC-derived frontal bone (Bildsoe et al., 2009). Our data indeed, reinforce the crucial role for *Twist1* in skeletogenic differentiation of the CNC and point to a differential effect of loss of *Twist1* in mesoderm-derived parietal bones. However, this observation deserves further investigation aimed to identify potential players in controlling the negative effect elicited postnatally by *Twist1* haploinsufficiency in frontal bone.

Taken together, our data point to a postnatal impact of *Twist1* haploinsufficiency in conferring a higher osteogenic ability to the mesoderm-derived parietal bone relative to the neural crest-derived frontal bone. Since, the coronal suture is comprised mostly of mesoderm-derived tissue (Jiang et al., 2002; Yoshida et al., 2008), our finding triggers interest to investigating whether a same unique *Fgf23* expression pattern, as that observed in wild-type and *Twist1*^+/−^ parietal bones, is also present between *Twist1*^+/−^ coronal synostotic and wild-type coronal patent sutures. A potential downregulation of *Fgf23* within the mesoderm tissue of *Twist*^+/−^ coronal suture could indeed induce synostosis.

*Fgf23* gene transcription in bone remains largely undefined, therefore the finding that either *Twist1* haploinsufficiency or its sh-mediated silencing suppressed the expression of *Fgf23* in mesoderm-derived parietal bone and osteoblasts is interesting and raises the question whether this outcome is a direct or indirect effect elicited by Twist1. Is Twist1 a transcriptional activator of *Fgf23* gene in parietal bones? Are there tissue-specific co-activators involved in the process? These are important and challenging questions that deserve to be addressed. Twist1 binds conserved *cis*-regulatory elements known as E-boxes present on gene promoters, triggering either transcriptional activation or repression of genes (Hamamori et al., 1997; Murre et al., 1989; Massari and Murre, 2000). Initial analysis of murine *Fgf23* gene have identified within its core promoter region E-Box motifs (CANNTG), thus suggesting that *Fgf23* could be a direct target of Twist1 (data not shown). This hypothesis finds support from our sh*Twist1*^+/−^ silencing experiments on POb showing that *Twist1* silencing recapitulated the expression pattern of *Fgf23* as well as that of specific *Twist1* markers initially identified by RNA-Seq analysis in *Twist1*^+/−^ parietal bones.

## Conclusion

In conclusion, from our study emerged two novel findings: first, *Twist1* haploinsufficiency selectively promotes a greater osteoskeletal ability and regeneration of mesoderm-derived calvarial bone, whilst negatively impacting neural crest-derived calvarial bone. A previous study has described that *TWIST-1* haploinsufficiency enhanced osteogenesis of osteoblast cells derived from parietal bones of patients Saethre–Chotzen syndrome, however, the study is missing the analysis of osteoblasts derived from a frontal bone (Camp et al., 2018). Thus, by focusing our analysis on two calvarial bones of different tissue origin, we have highlighted a dual role of *Twist1* haploinsufficiency. A second finding is the identification of a molecular mechanism though which *Twist1* haploinsufficiency triggers preferentially osteoskeletal induction. Herein, we provide substantial evidence that this occurs via downregulation of *Fgf23*, which is uniquely expressed at high threshold levels in wild-type parietal bone. The innate mechanism presented in this paper, through which *Twist1* haploinsufficiency preferentially triggers osteogenic induction of parietal bones may be beneficial to optimize treatments for skeletal regeneration, reconstruction and repair of mesoderm-derived bone, as well as to alleviate skeletal abnormalities caused by *Twist1* haploinsufficiency.

## Author Contributions

NQ designed and performed the experiments, analyzed the data, and wrote the manuscript. SS, NM, and SM performed the experiments. AR and ML edited the manuscript.

## Conflict of Interest Statement

The authors declare that the research was conducted in the absence of any commercial or financial relationships that could be construed as a potential conflict of interest.

## Footnotes

1 https://www.ncbi.nlm.nih.gov/geo/query/acc.cgi?acc=GSE119962

2 http://www.bioinformatics.babraham.ac.uk/projects/fastqc/

3 http://drpowell.github.io/vennt/

4 https://doi.org/10.1093/bioinformatics/bth349
