# Supplementary figures and images for: Twist1-Haploinsufficiency Selectively Enhances the Osteoskeletal Capacity of Mesoderm-Derived Parietal Bone Through Downregulation of Fgf23
Source: Front Physiol. 2018 Oct 15;9:1426. doi: 10.3389/fphys.2018.01426 (PMC6196243; doi:10.3389/fphys.2018.01426)

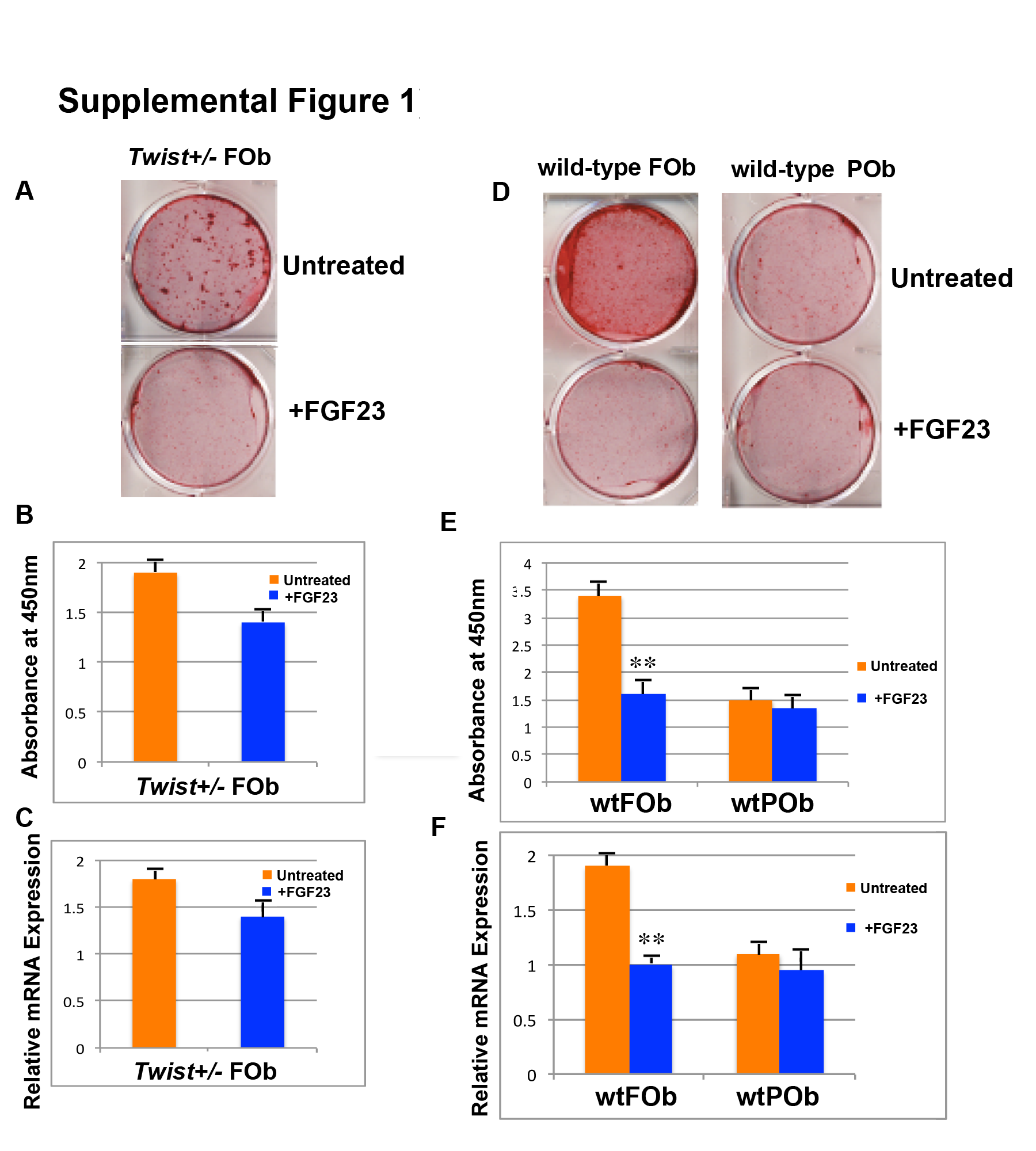

Supplement: Supplementary file 1 [file Image_1.TIF]

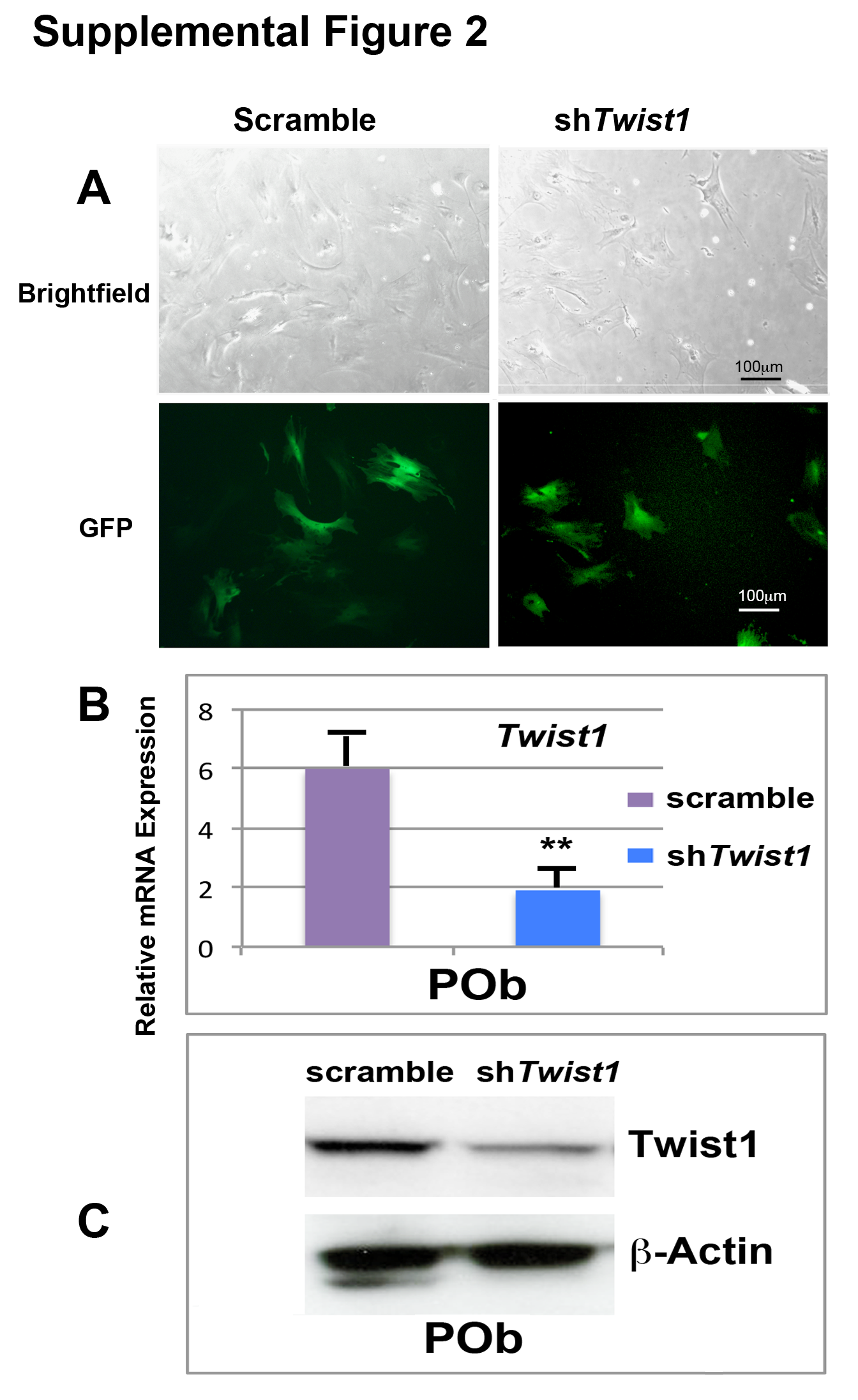

Supplement: Supplementary file 2 [file Image_2.TIF]

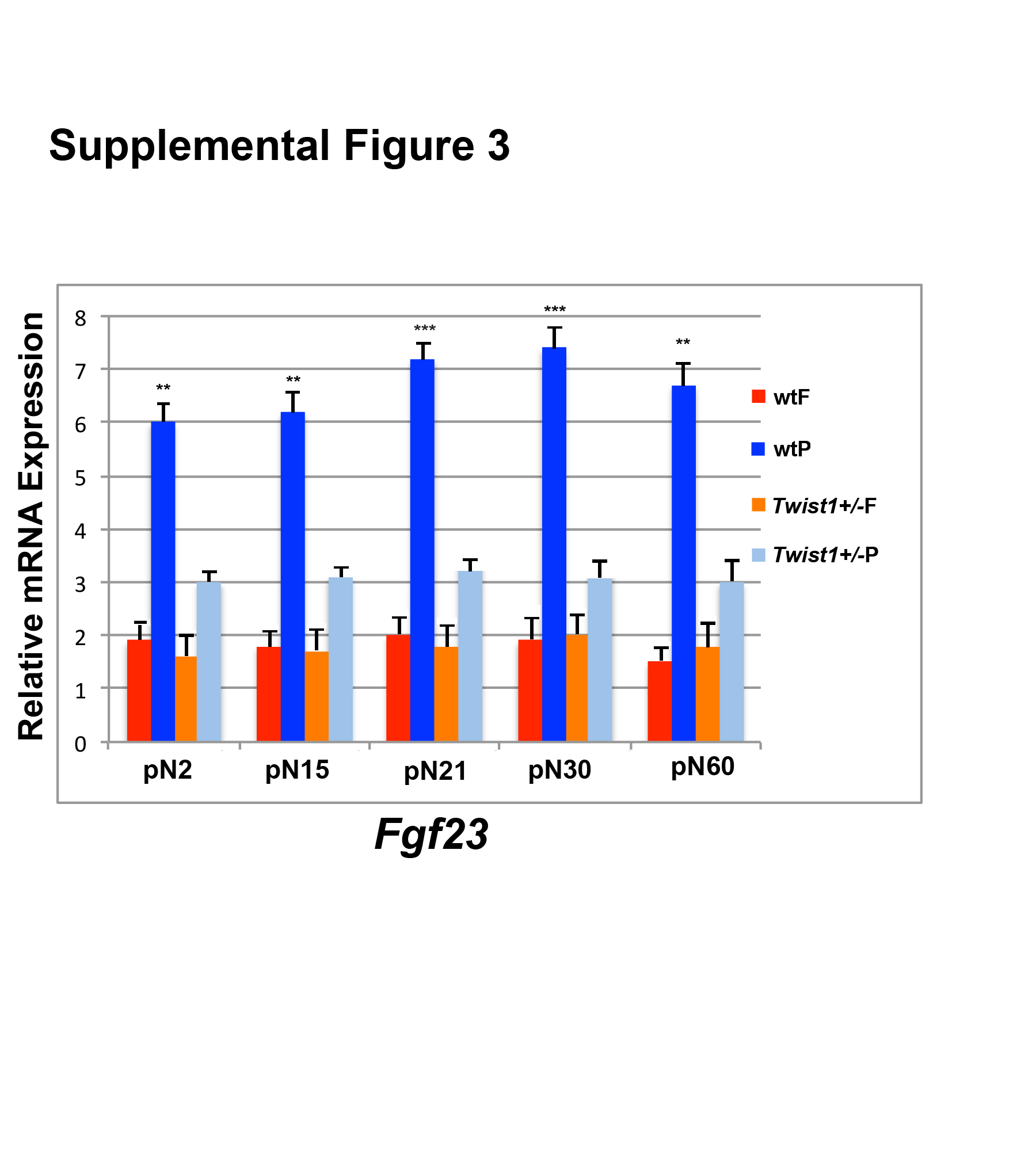

Supplement: Supplementary file 3 [file Image_3.TIF]
