# Supplementary material for: Twist1-Haploinsufficiency Selectively Enhances the Osteoskeletal Capacity of Mesoderm-Derived Parietal Bone Through Downregulation of Fgf23
Source: Front Physiol. 2018 Oct 15;9:1426. doi: 10.3389/fphys.2018.01426 (PMC6196243; doi:10.3389/fphys.2018.01426)
Supplement: Supplementary file 4 [file Table_1.docx]

**SUPPLEMENTAL FIGURE LEGENDS**

**Figure S1.** Effect of FGF23 treatment. **(A)** Alizarin red staining reveals a mild effect of FGF23 protein on Twist1^+/-^FOb. **(B)** Quantification of Alizarin red staining for the osteogenic assay shown in panel A. **(C)** RT-qPCR analysis of *Bglap* expression confirming the mild effect of FGF23 treatment. **(D)** Mineralization of extracellular matrix by Alizarin red staining shows a strong inhibition elicited by FGF23 treatment on wild-type FOb, whereas little on wild-type POb. **(E)** Quantification of Alizarin red staining for the assay in panel **D.** **(F)** RT-qPCR analysis of *Bglap* expression.

**Figure S2.** Efficiency of *Twist1* silencing in POb. **(A)** GFP immunofluorescence staining of scramble and sh*Twist1* POb co-transduced with GFP control lentiviral particles to assess the efficiency of transduction. Upper panel: bright field; Lower panel: immunofluorescence capturing. (Magnification 100x). **(B)** RT-qPCR analysis demonstrates the effective silencing of *Twist1* gene in POb transduced with sh*Twist1* lentiviral particle **(C)** Immunoblotting analysis performed using anti-Twist1 antibody validates at protein level the silencing.

**Figure S3. (A)** Time-course expression of *Fgf23* in wild-type and *Twist1* haploinsufficient frontal and parietal bones reveals a sustained upregulation of *Fgf23* thoroughly stages analyzed.
